# Characteristics and contraceptive outcomes of women seeking medical or surgical abortion in reproductive health clinics in Cambodia

**DOI:** 10.1186/s40834-019-0086-0

**Published:** 2019-05-16

**Authors:** Chris Smith, Rachel H. Scott, Caroline Free, Tansy Edwards

**Affiliations:** 1 Graduate School of Tropical Medicine and Global Health, Nagasaki University, Nagasaki, Japan; 2 Department of Population Health, London School of Hygiene & Tropical Medicine, London, UK; 3 MRC Tropical Epidemiology Group, London School of Hygiene & Tropical Medicine, London, UK

**Keywords:** Contraception, Post-abortion family planning, Post-abortion contraception, Medical abortion, Surgical abortion

## Abstract

**Background:**

Since the approval of medical abortion in Cambodia in 2010, the proportion of women reporting an abortion has increased. We describe the characteristics and contraceptive outcomes of women seeking medical abortion compared to surgical abortion at four reproductive health clinics in Cambodia.

**Methods:**

Secondary data analysis using data collected in the MObile Technology for Improved Family Planning (MOTIF) trial, a single blind, randomized trial of a personalized, mobile phone-based intervention designed to support post-abortion family planning in Cambodia. Baseline interviews were conducted after women had received post-abortion family planning counselling at the clinic, and follow-up interviews were conducted at 4 and 12 months. We used multivariable logistic regression to assess associations between abortion type and baseline characteristics, contraceptive uptake, repeat pregnancy and repeat abortion. We conducted an exploratory analysis to compare the timing of initiation of long-acting (LA) contraception between women having medical versus surgical abortion over the four-month post-abortion period.

**Results:**

Of the 500 women who participated in the trial, 41% had a medical abortion. In multivariate analyses, women undergoing medical abortion were more likely to be urban residents, have a higher level of education, be undecided or not intending to use family planning, and be undecided or intending to have another child. There was no association between type of abortion and contraceptive uptake, repeat pregnancy or repeat abortion. Women who had medical abortion initiated LA contraception post abortion later than women who had surgical abortion.

**Conclusions:**

Our results suggest women having a medical abortion in Cambodia have different baseline characteristics and had delayed uptake of contraception compared to women having a surgical abortion. However, we cannot draw conclusions on the direction of associations and causality. Further research is recommended to explore reasons for the observed findings with view to increasing access to abortion and post-abortion contraception.

## Introduction

It is estimated that globally around 25% of pregnancies resulted in induced surgical or medical abortion during the period 2010–2014, equating to an annual abortion rate of 35 per 1000 women aged 15–44. [1] In Cambodia in response to high maternal mortality, estimated at 900/100,000 live births, abortion laws were reformed in 1997 to allow abortion on request until the 12th week of pregnancy, and in certain circumstances during the second trimester. [2]. According to the 1997 law, abortions can only be provided by medical doctors, medical practitioners or midwives authorized by the Ministry of Health and only carried out in a hospital, health center, health clinic or maternity ward. [3] The medical abortion pack ‘Medabon’ containing mifepristone and misoprostol was approved by the Cambodia Ministry of Health in 2010 and made available in a restricted number of health facilities and pharmacies. [2] Medical abortion is available in clinic settings and from pharmacies and medical abortion packs have been distributed in Cambodia via social marketing and franchising programmes by non-governmental organisations such as Population Services Khmer. [4] Cambodian women also use other pharmaceutical products to induce an abortion, purchased from pharmacists, markets and drug sellers; typically unregistered combinations of mifepristone and misoprostol, sometimes described as “Chinese pills” [4, 5].

Sequential demographic and health surveys in Cambodia indicate that the proportion of women reporting having had an induced abortion in the previous 5 years increased from 4% in 2005 to 7% in 2014. [3, 6, 7] Following the approval of medical abortion in 2010, the proportion of women having medical rather than surgical abortion increased: from 31% in 2010 to 47% in 2014. [3, 7]

Women seeking abortion services often have an unmet need for family planning. Post-abortion family planning (PAFP) can reduce subsequent unplanned pregnancies and repeat abortion and was identified as a high-impact practice in family planning by a USAID technical advisory group in 2012. [8] Women are at risk of pregnancy soon after abortion; studies that have examined return to ovulation post-abortion show that this can occur around 2–3 weeks following the abortion, but earlier among some women. [9, 10] All contraceptive methods are safe to use in the immediate post-abortion period to reduce the risk of pregnancy, with the exception of the intra-uterine device (IUD), which can be inserted at the time of surgical abortion but in the case of medical abortion should be delayed until after the abortion is complete. [11–13] The type of abortion obtained may therefore affect uptake of post-abortion contraception or the contraceptive method chosen.

We describe characteristics and contraceptive outcomes of women seeking medical abortion vs. surgical abortion, using data collected in the MObile Technology for Improved Family Planning (MOTIF) trial. [14] The specific objectives are to compare: (1) baseline characteristics amongst women seeking medical versus surgical abortion in the MOTIF trial; (2) outcomes: contraception use, repeat pregnancy and abortion amongst women seeking medical versus surgical abortion in the trial; and (3) the timing of initiation of long-acting contraception between women having medical versus surgical abortion over the four-month post-abortion period.

## Methods

We analysed data collected during the MOTIF trial. The trial protocol, results and process evaluation are reported in separate manuscripts. [15, 16] In brief, the MOTIF trial was a single blind, randomized trial of a personalized, mobile phone-based intervention designed to support post-abortion contraception. Women were recruited by research assistants from four clinics in Cambodia during May to September 2013. The clinics in Phnom Penh (2), Battambang and Siem Reap served clients from urban and rural areas. On recruitment, women were asked whether they considered their residence to be urban or rural. Women were eligible for inclusion if they were aged 18 or older, attending for an induced abortion at the MSI clinic (either surgical or medical, using licensed medication), had a mobile phone primarily for their own use, reported not wanting to become pregnant and were willing to receive automated messages about contraception. Baseline interviews were conducted after women had received post-abortion family planning counselling at the clinic for the abortion visit, and follow-up interviews were conducted at 4 and 12 months. Follow-up was 86% at 4 months and 66% at 12 months. There was no evidence of differential loss to follow-up between women receiving medical compared to surgical abortion at four or 12 months. [17]

### Analysis

First, factors associated with medical versus surgical abortion in univariable analyses were identified, based on a likelihood ratio test (LRT) *p*-value< 0.1. A multivariable model was obtained from a stepwise model building process, whereby covariates were added to the multivariable model in turn, and the goodness of fit of the model with the additional parameter was compared to the previous model using a LRT. Covariates were retained in the multivariable model if the LRT *p*-value < 0.1. Covariates were removed if not associated with the outcome after adjustment for other covariates during this process.

Secondly, we assessed crude and adjusted associations between abortion method and effective contraceptive use, repeat pregnancy and abortion at 4 and 12-months using logistic regression. We define effective modern methods to be those associated with < 10% 12-month pregnancy rates, as commonly used: Oral Contraceptive (OC), injectable, implant, IUD, or permanent methods [18]. A participant was considered to be using an effective method if she reported that she: (i) was currently using the implant or IUD; (ii) had received the injectable within the previous 3 months; (iii) was sterilized (or partner was sterilized); or (iv) had taken an OC within 24 h of the interview or according to instructions. Long acting methods were defined as the IUD, implant or a permanent method. The pre-specified approach was to adjust for age, socio-economic status and residence a priori, as well whether the participant was allocated to receive the intervention or not. Access to motorised transport was used as a measure of socio-economic status, similar to the assessment of household wealth in the Cambodia DHS survey. [3] Age cut offs above and below 25 were selected to be consistent with MSI definitions of young women and our previous publications. [19] All participants who were allocated to the intervention and could be contacted received the intervention messages. Those who could not be contacted were considered lost to follow up and excluded from the analyses on contraceptive use, repeat pregnancy and repeat abortion.

Thirdly, we conducted an exploratory analysis to compare the timing of initiation of long-acting contraception between women having medical versus surgical abortion over the four-month post-abortion period. This analysis used data on weekly contraceptive use was obtained retrospectively at four-month follow-up.

Ethical approval for the MOTIF study was obtained from ethics committees at the London School of Hygiene and Tropical Medicine and Marie Stopes International and from the Cambodia Human Research ethics committee. All analyses were conducted using Stata, Version 15.

## Results

Of the 500 women who participated in the trial 41% had a medical abortion. Over two thirds of the sample were aged over 25, almost two thirds lived in rural areas, and the majority had access to motorized transport. Almost two thirds had secondary level education or above, and nearly all were married or living together. The full description of the sample is shown in Table 1. Table 2 shows the characteristics of women attending for medical versus surgical abortion. Univariable analyses suggested that younger age (*p* = 0.019), urban versus rural area of residence (*p* < 0.001), access to motorized transport (*p* = 0.058), higher level of education (*p* = 0.005), having no living children (*p* = 0.001), having mainly joint or mainly partner decision-making about contraception (*p* = 0.001), not intending or undecided about PAFP (*p* < 0.001), plans to have another child or undecided about more children (*p* < 0.001), were associated with increased odds of medical versus surgical abortion. Occupation was associated with type of abortion (*p* < 0.001). During development of a final multivariable model, age was included first, then factors identified in univariable analyses were added and evaluated for association with abortion type after adjustment in turn, first looking at socio-economic factors and then fertility and contraceptive related factors. The final model included level of education, PAPF intentions, fertility plans and area of residence. Age and number of children were no longer associated with type of abortion after adjustment for contraceptive and fertility plans. After adjustment for factors included in the final model, medical versus surgical abortion was more likely amongst those with more education, those without plans for or undecided about PAFP, those undecided or desiring a child in future and those living in urban areas. Whether contraceptive decisions were mainly made by a husband/partner or made jointly was only asked to those who were currently married. Of this group of 464 women, joint or partner contraceptive decision making was not strongly associated with type of abortion after adjustment for other factors in the final model for all women (*p* = 0.085). After adjustment for the other variables in the final model, occupation was not associated with abortion type (LRT *p* = 0.289).

**Table 1:** Characteristics of the study sample

	N	%
Type of abortion		
Medical	207	41
Surgical	293	59
Age group		
Age > 25	343	69
Age < 25	157	31
Residence		
Rural	321	64
Urban	179	36
Socio-economic status		
No access to motorised transport	65	13
Access to motorised transport	435	87
Level of Education		
None or primary	196	39
Some secondary or above	304	61
Marital status		
Never married or living together	29	6
Married/living together	464	93
Divorced/separated	7	1
# living children		
1 or more	353	71
0	147	29
# previous abortions		
0	299	60
1 or more	201	40
Previous contraception use		
Yes	299	60
No	201	40
Occupation		
Housewife	102	20
Factory worker	76	15
Entertainment worker	27	5
Farmer	32	6
Employed	99	20
Self-employed/own business	134	27
Casual work/casual labour	3	1
Student	17	3
Unemployed	9	2
vOther	1	0
Contraception decision-making^a^		
Mainly woman	96	21
Mainly husband/partner	78	17
Joint decision	290	63
Phone sharing		
Shares	241	48
Never shares	259	52
Disclosure of abortion to others^b^		
Yes	204	85
No	37	15
PAFP intentions		
Yes	187	37
No	42	8
Undecided	271	54
Fertility plans		
No more/none	139	28
Have a/another child	308	62
Undecided	53	11
Phone credit		
Sometimes	193	39
Usually	128	26
Always	179	36
Randomisation		
Intervention	249	50
Control	251	50

^a^36 missing values ^b^259 missing values

**Table 2:** Associations with type of abortion

	Surgical	Medical	Crude OR	LRT *p* value	Adjusted OR^a^	LRT *p* value
	*N* = 293	*N* = 207				
Age group						
Age > 25	213 (62%)	130 (38%)	1.00			
Age < 25	80 (51%)	77 (49%)	1.58 (1.08–2.31)	0.019		
Residence						
Rural	216 (67%)	105 (33%)	1.00			
Urban	77 (43%)	102 (57%)	2.73 (1.87–3.97)	< 0.001	2.18 (1.46–3.25)	< 0.001
Socio-economic status						
No access to motorised transport	45 (69%)	20 (31%)	1.00			
Access to motorised transport	248 (57%)	187 (43%)	1.70 (0.97–2.97)	0.058		
Level of Education						
None or primary	130 (66%)	66 (34%)	1.00			
Some secondary or above	163 (54%)	141 (46%)	1.70 (1.17–2.47)	0.005	1.45 (0.97–2.17)	0.068
Marital status						
Never married or living together	18 (62%)	11 (38%)	1.00			
Married/living together	271 (58%)	193 (42%)	1.17 (0.54–2.52)	0.924		
Divorced/separated	4 (57%)	3 (43%)	1.23 (0.23–6.55)			
# living children						
1 or more	223 (63%)	130 (37%)	1.00			
0	70 (48%)	77 (52%)	1.89 (1.28–2.78)	0.001		
# previous abortions						
0	171 (57%)	128 (43%)	1.00			
1 or more	122 (61%)	79 (39%)	0.87 (0.60–1.25)	0.435		
Previous contraception use						
Yes	180 (60%)	119 (40%)	1.00			
No	113 (56%)	88 (44%)	1.18 (0.82–1.69)	0.376		
Occupation						
Housewife	67 (66%)	35 (34%)	1.00			
Factory worker	35 (46%)	41 (54%)	2.24 (1.22–4.12)	< 0.001		
Entertainment worker	15 (56%)	12 (44%)	1.53 (0.65–3.63)			
Farmer	29 (91%)	3 (9%)	0.20 (0.06–0.70)			
Employed	57 (58%)	42 (42%)	1.41 (0.80–2.50)			
Self-employed/own business	78 (58%)	56 (42%)	1.37 (0.81–2.34)			
Casual work/casual labour	2 (67%)	1 (33%)	0.96 (0.08–10.93)			
Student	5 (29%)	12 (71%)	4.59 (1.50–14.09)			
Unemployed	4 (44%)	5 (56%)	2.39 (0.60–9.48)			
Other	1 (100%)	0 (0%)	1.00 (.-.)			
Contraception decision-making						
Mainly woman	71 (74%)	25 (26%)	1.00			
Mainly husband/partner	47 (60%)	31 (40%)	1.87 (0.98–3.56)	0.001		
Joint decision	154 (53%)	136 (47%)	2.51 (1.50–4.18)			
Phone sharing						
Shares	142 (59%)	99 (41%)	1.00			
Never shares	151 (58%)	108 (42%)	1.03 (0.72–1.46)	0.888		
Disclosure of abortion to others						
Yes	122 (60%)	82 (40%)	1.00			
No	20 (54%)	17 (46%)	1.26 (0.63–2.56)	0.515		
PAFP intentions						
Yes	135 (72%)	52 (28%)	1.00			
No	22 (52%)	20 (48%)	2.36 (1.19–4.68)	< 0.001	2.08 (1.02–4.23)	0.001
Undecided	136 (50%)	135 (50%)	2.58 (1.73–3.84)		2.18 (1.44–3.31)	
Fertility plans						
No more/none	105 (76%)	34 (24%)	1.00			
Have a/another child	162 (53%)	146 (47%)	2.78 (1.78–4.35)	< 0.001	2.07 (1.29–3.32)	0.007
Undecided	26 (49%)	27 (51%)	3.21 (1.65–6.22)		2.08 (1.03–4.18)	
Phone credit						
Sometimes	123 (64%)	70 (36%)	1.00			
Usually	67 (52%)	61 (48%)	1.60 (1.02–2.52)	0.120		
Always	103 (58%)	76 (42%)	1.30 (0.85–1.97)			

^a^Adjusted for factors included in the final model

Table 3 shows associations between abortion method and subsequent contraceptive use, repeat pregnancy and repeat abortion. Surgical abortion was associated with increased odds of using effective contraception at 4 months compared to medical abortion but this association did not remain after adjusting for confounding variables (aOR 1.44; 95%CI 0.95–2.18). There were no statistically significant differences in contraceptive use, repeat pregnancy or repeat abortion comparing women having medical versus surgical abortion, but it is acknowledged that power was low for these analyses.

**Table 3:** Associations between abortion method and subsequent contraception use, repeat pregnancy and abortion

Four month follow-up							12 month follow-up					
	No	Yes	Crude OR	*P* value	Adjusted OR*	*P* value	No	Yes	Crude OR	*P* value	Adjusted OR*	*P* value
Effective contraception use												
Medical	93 (52%)	85 (48%)	1.00				75 (54%)	63 (46%)	1.00			
Surgical	102 (40%)	151 (60%)	1.62 (1.10–2.38)	0.015	1.44 (0.95–2.18)	0.084	101 (53%)	89 (47%)	1.05 (0.68–1.63)	0.831	0.87 (0.55–1.39)	0.563
Long-acting contraception use												
Medical	146 (82%)	32 (18%)	1.00				113 (82%)	25 (18%)	1.00			
Surgical	205 (81%)	48 (19%)	1.07 (0.65–1.75)	0.794	0.87 (0.51–1.49)	0.611	154 (81%)	36 (19%)	1.06 (0.60–1.86)	0.848	0.84 (0.46–1.54)	0.573
Repeat pregnancy												
Medical	171 (97%)	6 (3%)	1.00				116 (84%)	22 (16%)	1.00			
Surgical	248 (98%)	5 (2%)	0.57 (0.17–1.91)	0.367	0.82 (0.23–2.86)	0.750	162 (85%)	28 (15%)	0.91 (0.50–1.67)	0.764	0.97 (0.51–1.84)	0.924
Repeat abortion												
Medical	176 (99%)	1 (1%)	1.00				127 (92%)	11 (8%)	1.00			
Surgical	251 (99%)	2 (1%)	1.40 (0.13–15.59)	0.783	2.04 (0.17–24.22)	0.572	182 (96%)	8 (4%)	0.51 (0.20–1.30)	0.157	0.50 (0.19–1.35)	0.172

*Adjusted for age, residence, socio-economic status and whether randomised

**Fig. 1:**
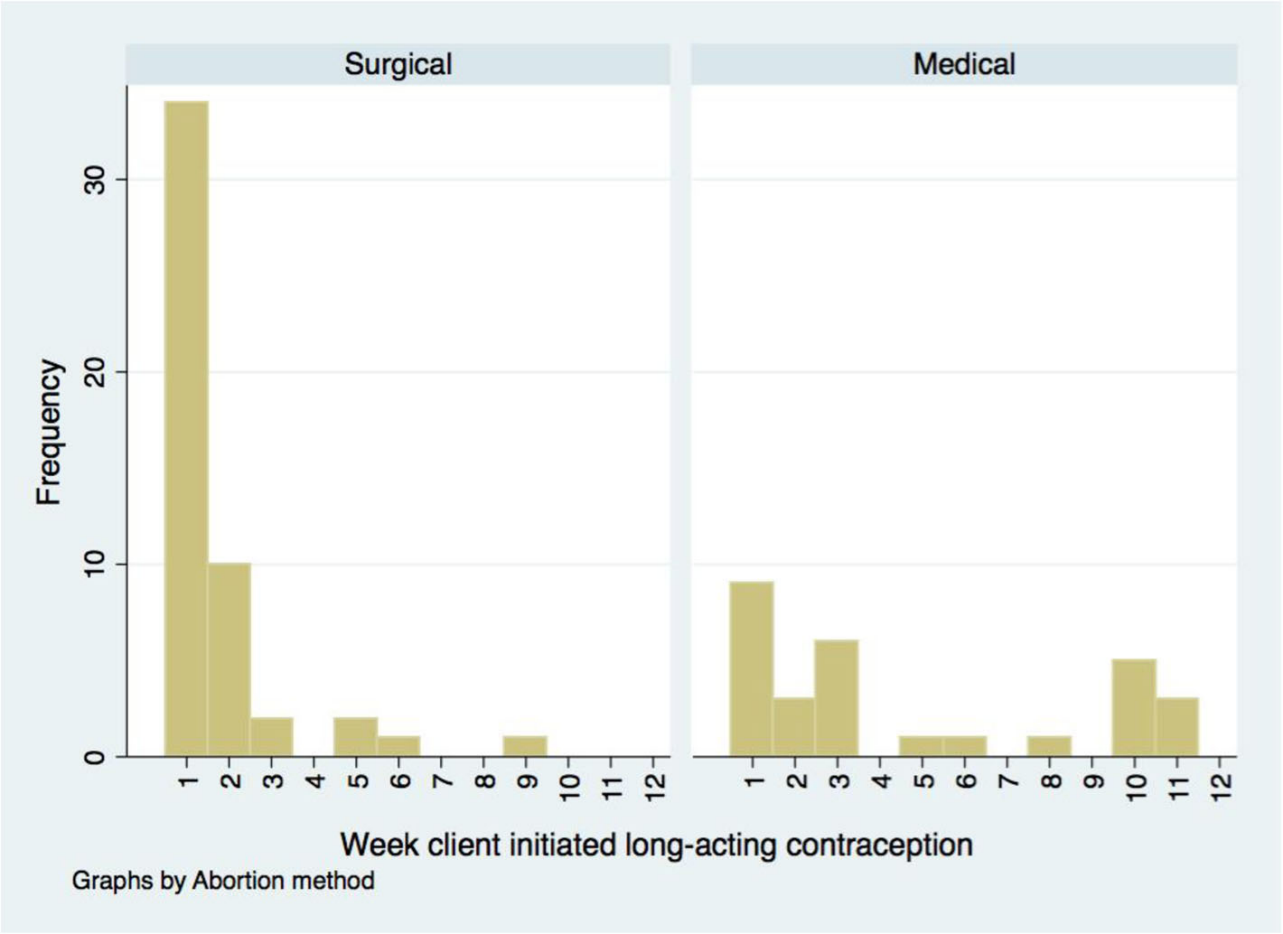
Initiation of long-acting contraception (IUD or implant) over 4-month post-abortion period *data obtained retrospectively at 4months asking participants about weekly contraceptive use. 50 women reported initiating long-acting contraceptive after surgical abortion, of whom 34 (68%) started during the first week. 29 women reported initiating long-acting contraception use after medical abortion, of whom 9 (31%) started during the first week

Figure 1 shows the timing of initiation of long-acting contraception over the four-month post-abortion period. Women having surgical abortion appear more likely to initiate long-acting contraception sooner compared to women having medical abortion; 50 women reported initiating long-acting contraceptive after surgical abortion, of whom 34 (68%) started during the first week. 29 women reported initiating long-acting contraception use after medical abortion, of whom nine (31%) started during the first week (*p* = 0.001). Of the 34 women who had a surgical abortion and started using long-acting contraception during the first week, 14 (41%) started using an IUD and 20 started using the implant. Of the nine women who had a medical abortion and started using long-acting contraception during the first week, two (22%) started using an IUD and seven started using the implant.

## Discussion

### Summary of main results

In this observational study using randomized controlled trial data, women having a medical abortion were more likely than those having a surgical abortion to be of urban residence, more educated, not planning to use or undecided about PAFP, and be planning to have another child or undecided. There was no association between type of abortion (medical vs. surgical) and use of effective contraception at 4 months, repeat pregnancy, or repeat abortion. Initiation of long-acting contraception within the first week was less likely with medical than surgical abortion.

### Strengths and weaknesses

To our knowledge, this is the first quantitative study reporting differences in characteristics of medical versus surgical abortion clients attending non-governmental health facilities in Cambodia, and complements trends in medical abortion use observed in the Demographic and Health Survey and previous qualitative studies in Cambodia and neighbouring Vietnam. [2, 5, 20] The study benefits from a sample size of 500 and a follow-up period of 12 months with good follow-up rates.

A key limitation of the study is the potential for residual and unmeasured confounding. It would have been particularly useful to have data on pregnancy duration. The 2014 Cambodian Demographic and Health Survey reported that use of surgical abortion increased with age and pregnancy duration [3, 7]. We also do not have information on provider behaviors – it is possible that providers may show bias in the type of abortion that they offer or recommend. Furthermore, we cannot draw conclusions on the direction of associations and causality.

A further limitation concerns the generalizability of the findings. These results reflect the NGO clinic population and may be different in public and private health facilities, and among women obtaining medical abortion from pharmacies. However, the proportions obtaining medical abortion are similar to 2013 data from MSI Cambodia. [14] In addition, the sample was restricted to women who had access to a mobile phone, although in Cambodia over 90% of women are estimated to own a mobile phone [21]. Future research should examine the needs of women using MA outside of the formal heath sector (e.g. from drug sellers) in terms of follow-up and PAFP. Further qualitative research could also consider how fertility intentions and provider biases might influence both the type of abortion that a woman has and subsequent use of PAFP.

Finally, the study was underpowered to examine differences in contraceptive and reproductive outcomes between women having medical and surgical abortions – although we found no evidence of a difference in PAFP uptake, repeat pregnancy or repeat abortion in this sample, a larger study may do.

### Discussion of the findings in context of existing research

Previous research has shown that a wide range of socio demographic variables as well as knowledge of abortion methods influence the uptake of medical abortion rather than surgical abortion. Our finding that rural women were less likely to choose MA are consistent with findings from Ethiopia, India, and Nepal but not Vietnam [22–25]. Our finding that educated women were more likely to use MA are in keeping with study findings in the USA and Nepal, but studies in Ethiopia and Vietnam did not find such an association [24–26]. To our knowledge no previous studies have reported the association between choice of MA and uncertainty about future contraceptive and fertility plans.

Access to a clinic is a barrier to obtaining abortion services and poor geographic access has been shown to be a barrier to women seeking other maternal health services in rural areas of Cambodia [27, 28]. Our finding that women living in rural areas are more likely to opt for surgical abortion rather than medical abortion could be due to the distance to clinic and the desire for greater certainty about the abortion being complete at the time of the clinic visit. It may also be that greater difficulties in access result in rural women attending later for medical abortion.

In this setting, women were offered a follow-up visit to check for post-abortion complications or a continuing pregnancy after both medical and surgical abortion as per national guidelines, [13] although routine data show that many women do not attend. [29] MSI Cambodia reports a 97.3% success rate for medical abortion. Self-assessment of the outcome of medical abortion is accurate, feasible and safe in low-resource settings. [30, 31] Enabling women to self-manage follow-up, as recommended in the WHO guidelines, [32] is likely to reduce barriers to medical abortion in urban and rural low-resource settings. [33].

Women with more education might be more confident in using medical abortion as it might be easier for them to read any written instructions provided and contact the clinic in case of a problem.

The associations between abortion type and contraception and fertility intentions could be explained by concerns about future fertility. Women who were undecided or planning on having more children (i.e. birth spacers rather than limiters) may have a preference for medical abortion if surgical abortion or contraception use is perceived as more harmful, potentially affecting the uterus and impacting fertility. [4]

Women undergoing medical abortion were no less likely than those undergoing surgical abortion to obtain post-abortion family planning (PAFP), including long-acting reversible contraception (LARC). All participants received standard care at the clinic prior to randomization, which included contraceptive counselling and the choice of a range of methods (e.g. pill, injectable and implant), all of which could be obtained on the day. It is unsurprising that women obtaining MA have lower uptake of IUD than women having surgical abortion as women could have an IUD inserted during surgical procedure, while women undergoing medical abortion have to attend and appointment for fitting 2 weeks later. It is perhaps surprising that women undergoing surgical abortion were not more likely to use LARC given the convenience of IUD insertion during the procedure, versus a repeat visit following medical abortion. However, LARC use is relatively low in Cambodia; roughly 2% of married women use the implant and 4% use the IUD compared to 9% who use the injectable, and 18% who use the contraceptive pill [3]. In contexts with a different baseline method mix, there might be greater differences in uptake of LARC between those undergoing medical and surgical abortion.

The findings did show that among women who started LARC methods post-abortion, women who obtained a medical abortion had a more delayed initiation. This may reflect the delay required between the procedure and the insertion of an IUD following medical abortion. This is supported by the finding that, among those who started LARC in the first week, the proportion starting to use an IUD (rather than implant) was higher among women who had a surgical abortion. Additionally, women having a medical abortion may have wished to have confirmation that the abortion was complete before starting to use the implant.

## Conclusion

Our results suggest women having a medical abortion in Cambodia are more likely to be educated and live in urban areas and had delayed uptake of contraception compared to women having a surgical abortion, although there was not strong evidence for a difference in contraceptive use at four or 12 month follow up. Our findings suggest there is a continuing need to improve access to abortion services, particularly in rural areas, as well as to take into account the factors influencing women’s choice of abortion method during the consultation. They also raise questions about whether routine follow-up is required after abortion. Enabling self-management of medical abortion follow up may increase women’s choice around the procedure by enabling women who would prefer medical abortion but cannot because of loss of earnings or distance barriers to do so Further research is recommended to increase access to abortion and post-abortion contraception.
